# Adaptive 3D Augmentation in StyleGAN2-ADA for High-Fidelity Lung Nodule Synthesis from Limited CT Volumes

**DOI:** 10.3390/s25247404

**Published:** 2025-12-05

**Authors:** Oleksandr Fedoruk, Konrad Klimaszewski, Michał Kruk

**Affiliations:** 1 Institute of Information Technology, Warsaw University of Life Sciences, 02-776 Warsaw, Poland; michal_kruk@sggw.edu.pl; 2 Department of Complex Systems, National Centre for Nuclear Research, 05-400 Otwock, Poland; konrad.klimaszewski@ncbj.gov.pl

**Keywords:** generative adversarial networks (GAN), 3D deep learning, adaptive discriminator augmentation (ADA), volumetric medical imaging, computed tomography (CT), medical image synthesis, lung nodules

## Abstract

Generative adversarial networks (GANs) typically require large datasets for effective training, which poses challenges for volumetric medical imaging tasks where data are scarce. This study addresses this limitation by extending adaptive discriminator augmentation (ADA) for three-dimensional (3D) StyleGAN2 to improve generative performance on limited volumetric data. The proposed 3D StyleGAN2-ADA redefines all 2D operations for volumetric processing and incorporates the full set of original augmentation techniques. Experiments are conducted on the NoduleMNIST3D dataset of lung CT scans containing 590 voxel-based samples across two classes. Two augmentation pipelines are evaluated—one using color-based transformations and another employing a comprehensive set of 3D augmentations including geometric, filtering, and corruption augmentations. Performance is compared against the same network and dataset without any augmentations at all by assessing generation quality with Kernel Inception Distance (KID) and 3D Structural Similarity Index Measure (SSIM). Results show that volumetric ADA substantially improves training stability and reduces the risk of a mode collapse, even under severe data constraints. A strong augmentation strategy improves the realism of generated 3D samples and better preserves anatomical structures relative to those without data augmentation. These findings demonstrate that adaptive 3D augmentations effectively enable high-quality synthetic medical image generation from extremely limited volumetric datasets. The source code and the weights of the networks are available in the GitHub repository.

## 1. Introduction

Computer vision in medical imaging is widely used to build decision-support systems for diagnosis and initial analysis [1,2]. There are many challenges in obtaining sufficient data and de-identifying patient data. Those problems become even more critical in terms of 3D (volumetric) data [3]. In this paper, we investigate the feasibility and effectiveness of applying StyleGAN2-ADA adapted for 3D datasets with a limited number of 3D images. Generative adversarial networks (GANs) are a machine learning framework in which two neural networks (generator, *G*, and discriminator, *D*) compete against each other in a zero-sum game. The goal of the discriminator is to distinguish real images from the synthetic images produced by the generator. The goal of the generator is to produce realistic images. The original GAN architecture was introduced in 2014 by Ian Goodfellow et al. [4] and has undergone multiple improvements and modifications since then. In this research we concentrate on StyleGAN2-ADA introduced by T. Karras et al. in 2020 [5], as one of its key features is the ability to successfully train on small datasets. Adaptive Discriminator Augmentation (ADA) is a training strategy that combats discriminator over-fitting. Rather than choosing a fixed augmentation pipeline, ADA adaptively adjusts the augmentation probability *P* based on the discriminator’s behavior. It monitors the proportion of real training images that receive positive discriminator outputs. If the proportion becomes too high, *P* is increased. If the proportion decreases, *P* is lowered accordingly.

The manuscript is organized as follows. Section 1 provides introduction and the overview of related works and the usage of the GAN in medical imaging. Section 2 describes the 3D dataset used for the experiments, 3D network architecture, implemented augmentations, and the training process. Additionally, in Section 2, we provide a brief explanation of the quality evaluation metrics, along with example results. Then in Section 3 we present the final findings and ideas for the future research. Our main contributions are as follows:

- StyleGAN2-ADA PyTorch adaptation for 3D data with all augmentation methods of the base implementation adapted to work with 3D data.
- An empirical evaluation of the adapted network across varying augmentation settings (full augmentation set, color-only augmentations, and no augmentations at all) for the balanced dataset containing 590 3D images of CT scans of lung nodules. The metrics used in evaluation are Kernel Inception Distance and 3D Structural Similarity Index Measure calculated on the generated and original objects.
- A comparative analysis using established image-synthesis metrics between three different augmentation scenarios.

### Related Works

The usage of a GAN to generate medical data is a promising field of research. Often combined with downstream tasks like classification and detection, it is applied to different types of data—such as brain tumor MR images [6], dermatoscopic images [7], retinal fundus data [8], chest X-rays [9,10,11], and colonoscopy frames [12]. The slice-wise approach is also often used due to small computational resources requirements, for example, in rib segmentation on X-ray slices [11] or synthetic CT reconstruction from MR slices [13]. The main challenge remains in combining the generated slices into the final 3D form while preserving medical features. Three-dimensional convolutional GANs are also widely used with different types of data, including applications in urogenital imaging [14] and hybrid augmentation pipelines for lesion classification [12]. The adaptation of StyleGAN2-ADA for 3D medical imaging was explored by Hong et al. in [15], but ADA itself was not used in their published experiments. Although a public repository [16] associated with that work provides a partial implementation of 3D augmentations, these augmentations were not employed in the paper, and only a limited subset of simple transformations is actually enabled in the code. Most geometric operations (e.g., flipping, rotation, translation, scaling, cutout) and advanced filtering augmentations are disabled or not adapted for 3D, leaving primarily basic color adjustments and noise injection. In contrast, our implementation is based on the official PyTorch codebase [17] and incorporates the full set of ADA mechanisms adapted for 3D.

The recent surge in applications of vision transformers and diffusion models to 3D medical imaging demonstrates promising developments in the field; however, data scarcity remains one of the most significant obstacles to future progress [6,18,19,20,21]. Diffusion models have shown strong potential in medical imaging, particularly for denoising, reconstruction, and synthetic data generation [22]. Their ability to capture complex distributions has been applied to MRI and CT, often outperforming GANs in sample fidelity [23]. Moreover, hybrids combining diffusion with vision transformers are emerging; for example, adversarial diffusion networks enhanced with Local Vision Transformers for MRI reconstruction [24] and transformer-based diffusion frameworks for segmentation [25]. However, both of those new architectures require vast training samples to fully realize their potential.

## 2. Materials and Methods

### 2.1. Dataset

We use NoduleMNIST3D [26] from MedMNIST benchmark dataset. The dataset contains 1633 objects representing computed tomography (CT) scans of lung nodules. The dataset is split into 3 subsets—training, containing 1158 objects; validation, containing 165 objects; and testing, containing 310 objects. The dataset is divided into two classes by the level of malignancy of the nodules. Class 0 (negative) contains nodules with malignancy levels 1 or 2; class 1 contains malignancy levels 4 or 5.

The training set of the NoduleMNIST3D dataset is not balanced—out of 1158 objects there are 863 objects of class 0 and 295 objects of class 1. The randomly selected 568 objects of class 0 were removed from the dataset. This serves two purposes—balancing the dataset and reducing the total amount of available training data to better simulate a limited data availability scenario.

The images are spatially normalized to 1 × 1 × 1 mm voxel spacing. A center-crop cube of $80^{3}$ voxels around each nodule was downsampled to $28^{3}$ and then upsampled to $64^{3}\;$ [27,28]. Due to memory constraints of the utilised NVIDIA A100 GPUs, for training we have downsampled them to $32^{3}$ size. Each image representing a nodule was stored as PyTorch tensor with shape of 32 × 32 × 32 and data-range 0–255.

**Figure 1:**
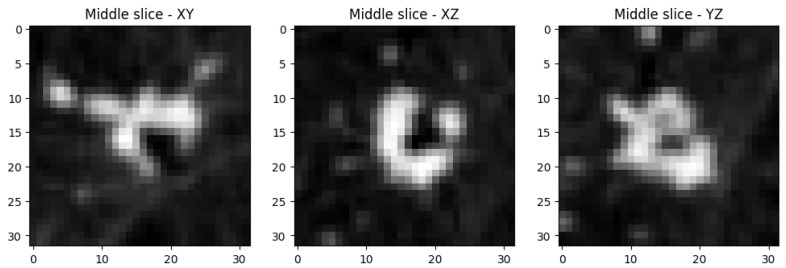
Middle slices along each principal axis of a lung-nodule volumetric object from the training dataset.

A total of 295 images of each class are used for training of the networks (590 training images in total). The middle slices of an example object from the dataset are represented in Figure 1.

### 2.2. Networks

Our solution is based on the official PyTorch implementation of StyleGAN2-ADA with original generator and discriminator architectures. We replaced all 2D convolution layers, resampling and gradfix operations with 3D counterparts. We closely follow the original implementation in selection of architecture details like convolutional kernel sizes, stride configurations, weights demodulation, and regularization, expanding to third dimension when necessary retaining observed symmetry. The mapping network remained the same as in the base 2D implementation. The StyleGAN2 loss function was adjusted to work with 3D images instead of 2D ones. Depth dimension (*D*) was added to R1 gradient penalty. Noise normalization in path length regularization was modified to divide by $\sqrt{D\cdot H\cdot W}$ instead of $\sqrt{H\cdot W}$.

The base implementation provides heuristics to decide which PyTorch memory format should be used (*contiguous* or *channels last*). We removed these heuristics in favor of *channels last* as the default memory format for both forward and backward passes.

The source code of the implementation used during the experiments is available at the GitHub repository: https://github.com/fedorukol/3d-stylegan2-ada (accessed on 1 December 2025).

### 2.3. Augmentations

All two-dimensional methods of the augmentation pipeline from the base implementation were adapted to operate on volumetric data. These include the following:

- **Pixel-level editing**—horizontal flipping along the sagittal axis, random rotations in the axial plane, and integer translations along the sagittal, coronal, and axial axes.
- **Geometric transformations**—isotropic scaling, arbitrary rotations, anisotropic scaling, and fractional translations.
- **Color transformations**—adjustments to brightness, saturation, and contrast; luma inversion; and hue rotation.
- **Image-space filtering**—filtering by amplification or suppression of the frequency content in different bands.
- **Image corruptions**—cutouts of parts of the images and application of Gaussian noise.

### 2.4. Training

We compare three augmentations pipelines: no augmentations at all, color-only augmentations, and strong augmentations with all transformations enabled. All three networks are trained with the same set of hyperparameters that were selected for providing the best results after several experimental runs. Each network is trained to generate two classes from the original dataset.

The generator and discriminator are trained using the Adam optimizer with learning rates set to 0.002 and parameters $\beta_{1}=0.9$, $\beta_{2}=0.999$. Following the recommendation in the StyleGAN2-ADA publication, the value for $R_{1}$ regularization is selected empirically by testing multiple values. It was set to 10 initially; however, the network produced blurry images. The value was then reduced to 5, 1, 0.8, and 0.6, with 0.6 used in the final training. A very small value (0.1) was also tested, but the network failed to train and produced random noise without signs of improvement. The batch size is set to 16, and the ADA target to 0.7.

All networks are trained for 300 kimg, where kimg denotes the number of thousands of images processed over subsequent training epochs. In other words, the models see a total of 300,000 images. This training schedule requires approximately 36 h on an NVIDIA A100 GPU.

### 2.5. Quality Evaluation and Metrics

We use several image quality metrics to assess the quality of the produced results. Kernel Inception Distance (KID) [29] measures the squared Maximum Mean Discrepancy (MMD) between feature representations of real and generated images extracted from a pretrained Inception network, using a polynomial kernel. Lower KID values indicate that the distribution of generated images is closer to that of the real images. In our experiments, KID is calculated on the middle-depth slices of the images for each tick (which corresponds to approximately 4000 images seen by the discriminator of the network).

The best epoch for a given training process is picked on the basis of the KID curve smoothed with a moving-average filter. We compute a moving average over a fixed window of 5 checkpoints with 5% tolerance to obtain a more stable estimate of model performance. The epoch corresponding to the minimum of this smoothed KID curve is then selected as the best model snapshot, providing a robust criterion that reduces sensitivity to metric fluctuations and mitigates the risk of overfitting late in training.

For the selected network state snapshot, we evaluate each strategy based on 3D Structural Similarity Index Measure (SSIM) [30], a metric commonly used in the literature [15]. We use a cubic Gaussian window of size 11×11×11 with standard deviation σ=1.5 in all three spatial dimensions, and the standard SSIM stability constants $C_{1}={\left(0.01L\right)}^{2}$ and $C_{2}={\left(0.03L\right)}^{2}$ with L=1.

SSIM quantifies how structurally similar two samples are by comparing their local patterns of intensity, contrast, and texture within a 3D neighborhood window. A higher SSIM value indicates that the two volumes share more consistent spatial structure and therefore appear more alike [31]. To access the similarity between the original and generated nodules we calculate 3D SSIM for each pair of original objects from the training set. We also calculate it for each pair between original and generated objects. The calculated values are plotted on the histograms where bigger overlap means more similar objects.

## 3. Results

### 3.1. Findings

**Figure 2:**
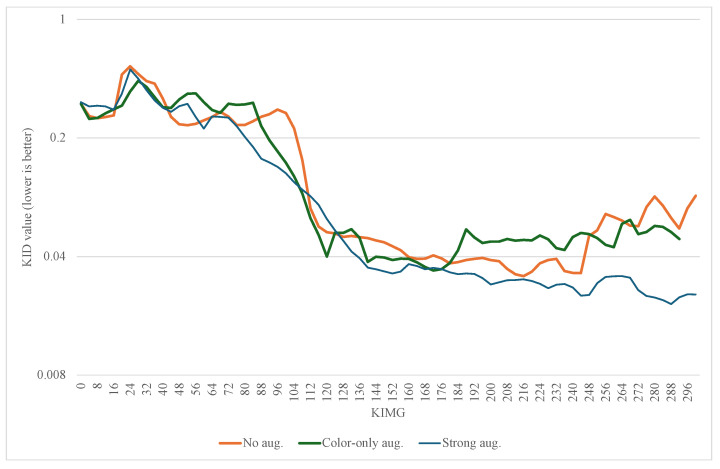
Mean value of KID for class 0 and class 1.

**Figure 3:**
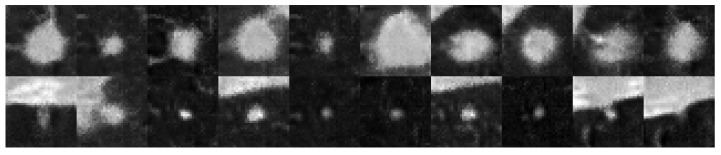
Middle slices of a lung nodule volumetric object generated by the network without ADA enabled. (**Top row**) contains images of the Class 0, (**bottom row**) contains images of the Class 1.

We observe that stronger augmentation ensures a better level of training stability in the 3D-adapted network. While color augmentation pipeline demonstrates a promising start, it is not enough to continue improving the generator as it is visible in Figure 2. After around 120,000 images shown to discriminator, the quality of generated samples starts to reduce. A similar scenario happens with the training without any augmentations. The KID value steadily decreases until a point where the discriminator starts to overfit and the generator starts to produce noisy images that are frozen into a small set of outputs, which could indicate an early stages of mode collapse. This behavior is visible at Figure 3 where most of the objects contain a similar single nodule.

**Figure 4:**
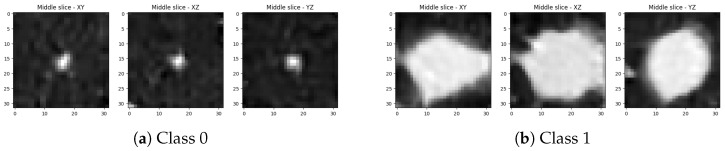
Middle slices along each principal axis for two lung-nodule volumetric objects generated by the best snapshot of the adapted StyleGAN2-ADA without augmentations: (**a**,**b**) show two representative examples.

**Figure 5:**
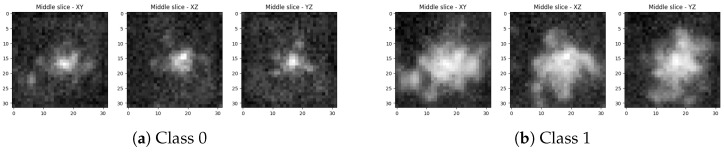
Middle slices along each principal axis for two lung-nodule volumetric objects generated by the best snapshot of the adapted StyleGAN2-ADA with color-only augmentations: (**a**,**b**) show two representative examples.

**Figure 6:**
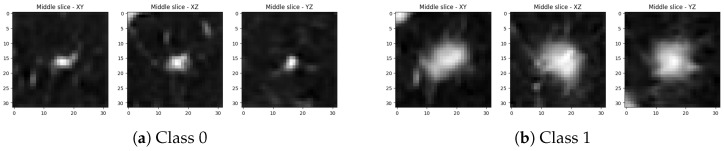
Middle slices along each principal axis for two lung-nodule volumetric objects generated by the best snapshot of the adapted StyleGAN2-ADA with strong augmentations: (**a**,**b**) show two representative examples.

The examples of the objects of both classes generated with the best snapshots of the networks without any, color-only, and strong augmentations are displayed on Figure 4, Figure 5, and Figure 6, respectively.

The evolution of the KID metrics for the no augmentation, color-only augmentation, and the strong augmentation is presented in Figure 2.

**Figure 7:**
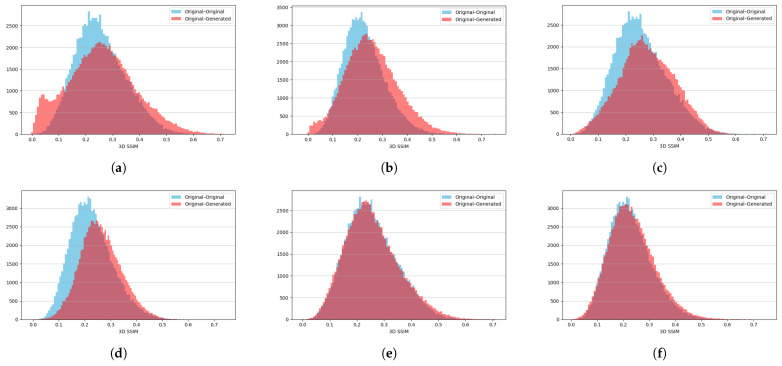
Three-dimensional SSIM histograms for the two object classes under three different training settings: (**a**,**b**) No augmentations for Class 0 and Class 1. (**c**,**d**) Color-only augmentation for Class 0 and Class 1. (**e**,**f**) Strong augmentation for Class 0 and Class 1.

The histograms of SSIM values for no augmentation, color-only augmentation, and the strong augmentation are presented in (Figure 7a,b), the 3D adapted network with color-only augmentation (Figure 7c,d), and the 3D adapted network with strong augmentation (Figure 7e,f), respectively.

### 3.2. Discussion

We introduce for the first time, a full set of 3D augmentation methods for ADA and successfully use it on limited medical imaging dataset. We demonstrate that the application of full set of augmentation techniques used in the 3D ADA pipeline makes it possible to train volumetric GANs on very small datasets. Moreover, it allows avoiding overfitting and mode collapse which are the main reasons why GANs are harder to train on small datasets. The styling mechanism incorporated into the StyleGAN architectures allows successful training for both classes and generates diverse and realistic output.

While achieved results are promising, there are several important topics to be considered. Full augmentation pipeline with all possible augmentation mechanisms could not be applied to every dataset—some volumetric features are not compatible with some augmentations. For example, data flips could not be used with chest PET scans, as they risk producing images in which the heart (and other organs) appear in anatomically incorrect positions [32]. This limitation reduces possible cases where such an approach could be applied.

Important limitation of our current implementation is its memory footprint. Due to memory constraints of the NVIDIA A100 GPUs, we reduced the image size considered in the research to $32^{3}$. However, with increasing sizes of VRAM in recent GPU architectures and possible optimizations to our implementation the proposed approach could be applied to larger resolutions.

### 3.3. Future Research

The quality of the generated images may be constrained when compared with more recent architectures such as vision transformers, which remain computationally demanding and particularly challenging to scale in three-dimensional settings. Future research will focus on extending the present findings to such architectures, examining diffusion-based models and/or vision transformers that offer higher generative quality but with reduced computational efficiency [33], and further reducing the cost of generating volumetric objects under low-data conditions.

Introduction of diffusion models and visual transformers opened new ways of graphical data generation with great results. We believe it is possible to incorporate achievements of those architectures in volumetric low-data scenarios by combining them with the introduced ADA methods. Finally, we plan to include downstream tasks like classification to additionally validate the findings.

## Data Availability

Publicly available datasets were analyzed in this study. We used the NoduleMNIST3D dataset from the MedMNIST collection, which is openly accessible at https://medmnist.com/ (accessed on 1 December 2025).
